# Examining the virulence of *Candida albicans* transcription factor mutants using *Galleria mellonella* and mouse infection models

**DOI:** 10.3389/fmicb.2015.00367

**Published:** 2015-05-05

**Authors:** Sara Amorim-Vaz, Eric Delarze, Françoise Ischer, Dominique Sanglard, Alix T Coste

**Affiliations:** Institute of Microbiology, University of Lausanne and University Hospital of LausanneLausanne, Switzerland

**Keywords:** *Candida albicans*, transcription factors, virulence, mice Galleria mellonella

## Abstract

The aim of the present study was to identify *Candida albicans* transcription factors (TFs) involved in virulence. Although mice are considered the gold-standard model to study fungal virulence, mini-host infection models have been increasingly used. Here, barcoded TF mutants were first screened in mice by pools of strains and fungal burdens (FBs) quantified in kidneys. Mutants of unannotated genes which generated a kidney FB significantly different from that of wild-type were selected and individually examined in *Galleria mellonella*. In addition, mutants that could not be detected in mice were also tested in *G. mellonella*. Only 25% of these mutants displayed matching phenotypes in both hosts, highlighting a significant discrepancy between the two models. To address the basis of this difference (pool or host effects), a set of 19 mutants tested in *G. mellonella* were also injected individually into mice. Matching FB phenotypes were observed in 50% of the cases, highlighting the bias due to host effects. In contrast, 33.4% concordance was observed between pool and single strain infections in mice, thereby highlighting the bias introduced by the “pool effect.” After filtering the results obtained from the two infection models, mutants for *MBF1* and *ZCF6* were selected. Independent marker-free mutants were subsequently tested in both hosts to validate previous results. The *MBF1* mutant showed impaired infection in both models, while the *ZCF6* mutant was only significant in mice infections. The two mutants showed no obvious *in vitro* phenotypes compared with the wild-type, indicating that these genes might be specifically involved in *in vivo* adapt

## Introduction

*Candida albicans* is a common commensal microorganism that persists on the mucosal surfaces of the human gastrointestinal tract and is also the most frequent human fungal pathogen (Odds, 1988). Superficial infections of the skin and mucosa are the most common diseases associated with this fungus. However, systemic infections (candidemia and invasive candidiasis) can occur in patients with compromised immunity. Despite several treatment possibilities, the mortality rates associated with these infections remain high, reaching 50% (Falagas et al., 2006; Leroy et al., 2009; Pfaller and Diekema, 2010). Thus, a better understanding of the fungal biology, particularly the host-fungal crosstalk during systemic infection, is needed for the development of new and more effective therapies.

*Candida albicans* adapts to drastically different environments, and this characteristic is crucial for the ability of these pathogens to cause invasive disease when transmitted from mucosal surfaces to the blood and internal organs. Transcription factors (TFs) are necessary to this process because these proteins mediate the rapid integration of external signals and the metabolic reprogramming to facilitate adaptation to the environmental conditions (Sellam et al., 2014). Accordingly, TFs are interesting starting points for the characterization of *C. albicans* biology, particularly virulence traits and drug resistance mechanisms.

Previous studies have attempted to explore *C. albicans* biology using different approaches. One valid possibility for understanding the role of *C. albicans* TFs is to identify orthologs in *Saccharomyces cerevisiae*. However, the evolution of the two organisms has resulted in extensive transcriptional rewiring (Rokas, 2006; Martchenko et al., 2007; Rokas and Hittinger, 2007; Baker et al., 2013), suggesting that homologous TFs might have different functions in the two yeasts. Alternatively, a commonly used approach is to generate mutant strains for genes encoding TFs and screen these strains for *in vitro* and/or *in vivo* phenotypes. The latter approach is particularly interesting in the context of virulence because these approaches generate information on the behavior of pathogens within a living organism (Noble et al., 2010; Vandeputte et al., 2011; Perez et al., 2013). Another approach is to observe the phenotypes of strains carrying hyperactive TF alleles produced through artificial activation (Devaux et al., 2001; Schillig and Morschhauser, 2013) and artificial TF overexpression (Devaux et al., 2001; Chauvel et al., 2012). Despite these efforts, many *C. albicans* regulatory circuits remain poorly understood.

Although mouse models confer many advantages for investigating fungal pathogenesis, these gold standard models have also been associated with some disadvantages, as mouse experiments are costly, logistically challenging, time-consuming and ethically delicate. To facilitate *in vivo* experimentation, a number of insect models have recently been developed for use with fungal pathogens, such as the larvae of the insect species *Galleria mellonella* (Cotter et al., 2000). These larvae have been increasingly used to study fungal virulence and antifungal drug activity (Brennan et al., 2002; Mylonakis et al., 2005; Coleman et al., 2011; Thomaz et al., 2013; Favre-Godal et al., 2014). The results obtained from this insect model were consistent with, first, those obtained from the mouse systemic model of infection but for a small number of mutants (Brennan et al., 2002; reviewed in Coste and Amorim-Vaz, 2015) and, second, with data on the pathogenicity of *C. albicans* strains in human patients (Cotter et al., 2000). The larvae can be maintained between 12 and 37∘C, facilitating the study of temperature-related virulence traits. The immune system of these insects can be compared with the innate immunity of mammals, and the larvae immune response to microorganisms can be assessed based on antimicrobial peptide (AMP) production or hemocyte counts (reviewed in Coste and Amorim-Vaz, 2015). Considering these advantages, *G. mellonella* is an interesting model for the large-scale screening of fungal pathogens and antifungal drugs, thereby narrowing the number of promising candidates for testing in mice.

In recent years, we initiated the *in vivo* screening of a *C. albicans* TF mutant collection using a mouse systemic model of infection to identify factors crucial for *C. albicans* virulence (Vandeputte et al., 2011). In a previous study, we focused on 77 mutants of the zinc cluster (Zn_2_Cys_6_) family of TFs (Vandeputte et al., 2011), characterized by the conserved motif CX_2_CX_6_CX_5-12_CX_2_CX_6-8_C and specific to the fungal kingdom (MacPherson et al., 2006), revealing a single TF, Zcf13, which was important for *C. albicans* infection in a mouse model of systemic infection (Vandeputte et al., 2011).

In the present study, we screened 157 mutants for other classes of TFs using two different animal models of infection and a new statistical analysis of previously obtained results. First, out of the total of 234 TF mutants injected into mice by pools and assessed for fungal burden (FB) in mice kidneys, we identified 47 mutants with increased or decreased tissue fungal loads, out of which 39 mutants were not previously known to play a role in virulence. These 39 mutants, along with 12 undetected mutants in the mice kidneys, were next tested in single strain infections. For ethical and financial reasons, the individual screening of these 51 mutants was performed using the alternative animal model *G. mellonella*. Thus, for the first time, two animal models, mouse and *G. mellonella*, were used in parallel to compare the FBs of a large number of mutants with yet unknown phenotypes. The results suggested that *G. mellonella* represents a useful, rapid and valid model to detect mutants with important defects in systemic host infection. Using this approach, two TF mutants with altered host infection capacities were identified.

## Materials and Methods

### Strains and Media

The *C. albicans* strains used in this study were obtained from a collection of TF mutants available at the Fungal Genetic Stock Center^1^ as previously described (Vandeputte et al., 2012). Each TF mutant from this collection was transformed with a plasmid containing a barcode as previously described (Vandeputte et al., 2011) and renamed BCYi for “BarCoded Yeast number i” (File S1). *C. albicans* strains were grown in complete YEPD medium [1% Yeast Extract (Difco), 2% Bacto Peptone, (Difco Laboratories, Basel, Switzerland) and 2% Dextrose (Fluka, Buchs, Switzerland)], supplemented or not with 200 μg/ml nourseothricin, or minimal medium YNB (Yeast Nitrogen Base) (Difco), containing 2% glucose (Fluka). For growth on solid media, 2% agar (Difco) was added. *Escherichia coli* DH5α was used as a host for plasmid construction and propagation. DH5α was grown in LB (Luria-Bertani broth) or on LB plates, supplemented with ampicillin (0.1 mg/ml) when required.

### Primers and Plasmids

The primers used in this study are listed in Supplementary Table S1, and the plasmids are listed in Supplementary Table S2.

### Yeast Transformation

Yeast transformation was performed as previously described (Sanglard et al., 1996).

### Construction of Deletion Mutants and Revertant Strains with Nourseothricin Resistance Cassettes

SC5314 was used as the parental strain to construct all marker-free, full ORF deletion mutants. The 5′- and 3′-flanking regions (500 bp) of each targeted gene were PCR amplified using the primers listed in Supplementary Table S1. The resulting fragments were introduced into pSFS2A (Reuss et al., 2004; see Supplementary Table S2). This plasmid contains the *SAT1*-flipping cassette, including *SAT1* (conferring resistance to nourseothricin), which is controlled through the *ACT1* promoter, and recombinase (*FLP*), which is controlled through a maltose-inducible promoter, and these genes were flanked by recombinase recognition target (FRT) sequences (Reuss et al., 2004). The resulting deletion plasmids are listed in Supplementary Table S2. The first allele of targeted TFs was deleted using pSV3 digested with KpnI and SacI (for *ZCF6*), pAC256 digested with KpnI and SacI (for *ZCF13*), and pAC286 digested with ApaI and SacI (for *MBF1*). Transformants from SC5314 were selected on YEPD containing nourseothricin (200 μg/ml). After induction of the recombinase in YEP medium containing 2% maltose for 4 h, nourseothricin-susceptible clones that underwent *SAT1* flipping were selected on YEPD agar plates containing only 15 μg/ml nourseothricin after 24 h incubation. At this drug concentration, susceptible *C. albicans* cells are still able to grow but much more slowly than resistant cells. These procedures result in the deletion of the targeted genes while maintaining intact 5′- and 3′- flanking regions. The second TF alleles of *ZCF6* and *MBF1* were subsequently deleted using the same deletion cassette. For *ZCF13*, the second allele was deleted through transformation with pAC245 digested with KpnI and SacI, resulting in a second deletion cassette internal to the first cassette. Transformants were selected on YEPD-nourseothricin (200 μg/ml), and examined using PCR to confirm the absence of wild-type alleles. Transformants without wild-type alleles were grown in YEP medium containing maltose (2%) for 4 h, and nourseothricin-susceptible clones were selected on YEPD agar plates containing only 15 μg/ml nourseothricin after 24 h incubation. To construct revertant strains, the wild-type alleles and corresponding 5′-flanking regions (500 bp) were PCR amplified from SC5314 using the primers listed in Supplementary Table S1. The resulting fragments were introduced into plasmids containing deletion cassettes of the corresponding TF genes, replacing the previously inserted 5′-flanking region, thus maintaining 3′-flanking regions and reconstituting wild-type alleles. Plasmids pSV4-1 and pSV4-2, containing the two different *ZCF6* wild-type alleles, were digested with KpnI and SacI. pAC291, containing the *MBF1* wild-type allele, was digested with ApaI and SacI. Plasmid pAC253, containing the *ZCF13* wild-type allele, was digested with KpnI and SacI. The resulting fragments were transformed into the corresponding mutant strains, replacing one of the mutated alleles through homologous recombination. The resulting strains are listed in Supplementary Table S3.

### Mice Experiments and Ethics Statement

All animal experiments were performed at the University Hospital Center of Lausanne with approval through the Institutional Animal Use Committee, Affaires Vétérinaires du Canton de Vaud, Switzerland (authorization n∘ 1734.2 and 1734.3), according to decree 18 of the federal law on animal protection. For all mice experiments, female BALB/c mice (6 weeks-old; Charles River France) were housed in ventilated cages with free access to food and water. The strains were grown in individual tubes for 16 h under agitation at 30∘C in YNB or YEPD medium. Each strain was subsequently diluted 100-fold in YEPD medium and grown overnight under agitation at 30∘C. Overnight cultures were washed twice with PBS and resuspended in 5 ml PBS. The concentration of each culture was measured through optical density, and each strain was diluted in PBS to the desired concentration.

The mice were injected with pooled fungal strains as previously described (Vandeputte et al., 2011), and the statistical analysis of the results was re-evaluated. Briefly, the mice were injected with pools of 10 *C. albicans* strains (wild-type, *cmp1*Δ/Δ and 8 other mutants), and each strain carried a specific barcode. DNA was extracted from the kidneys at 3 days post-infection (dpi) and from an *in vitro* culture after 24 h. Each mutant was quantified through qPCR targeting the barcode (see below). In the present study, we considered the variability in the efficacy of qPCR reactions for the different barcodes in the mutant strains (Supplementary Table S4). Thus, Mann–Whitney tests were performed to compare the FB scores of a given mutant with the score of a wild-type strain carrying the same barcode [see Quantitative PCR (qPCR) and Normalization].

For colony-forming unit (CFU) experiments with single strain infections, the mice were injected through the lateral tail vein with 250 μl of a cell suspension containing 8 × 10^5^ cells/ml. At three dpi, the kidneys were recovered, and the CFU were determined as previously described (Vandeputte et al., 2011). The mutants were tested in groups of five mice, once or twice (Experiment 1 and Experiment 2, see File S1). Statistical analyses of the differences between CFU values were performed using the Mann–Whitney test with a *p* ≤ 0.05 threshold. Supplementary Table S5 shows the correlation between results in the two subsets of mouse experiments. Additional statistical analyses with a *p* ≤ 0.05 threshold or using the False Discovery Rate (FDR = 0.05) according to Benjamini and Hochberg (1995) were also performed (Supplementary Table S5, File Table S1).

For survival experiments with single strain infections, groups of 7–10 mice were used. The mice were injected through the lateral tail vein with 250 μl of a cell suspension containing 2 × 10^6^ cells/ml. The weight and health of the animals were monitored daily. The post-infection day of natural death or euthanasia of moribund animals was recorded for each mouse. Survival experiments were terminated at 15 days after infection. The survival experiments were repeated twice for *zcf6*Δ/Δ and *zcf13*Δ/Δ mutants and three times for the *mbf1*Δ/Δ mutant. Statistical analyses of the survival data were performed using the log-rank (Mantel–Cox) test.

### Preparation of *C. albicans* DNA from Mice Kidneys

*Candida albicans* DNA was obtained from mouse kidneys as previously described (Vandeputte et al., 2011).

### Quantitative PCR (qPCR) and Normalization

Detection and quantification of each strain obtained from mouse kidneys infected with a pool of strains was performed through qPCR based on the barcode corresponding to each strain, as previously described (Vandeputte et al., 2011). Briefly, qPCR was performed using DNA extracted from the kidneys of infected and non-infected mice. The results obtained from the non-infected control mice were used to define the noise level. Pools of strains for which the C_T_ for signature-tagged mutagenesis barcode 6 (STM6; wild-type strain) was higher than that of the non-infected mice were rejected, as this result suggests that the *C. albicans* DNA extraction failed. These samples were excluded from further analysis. Ten standard curves were constructed, using 10-fold serial dilutions (10^5^–10^1^ copies) of the ten CIp30-STM plasmids and used to determine the copy number (Qx) of the barcode of each strain, either extracted from mouse kidneys infected with a pool of strains (Qx_INVIV O_) or from the same pool of strains grown *in vitro* (Qx_INVITRO_). The “*in vivo/in vitro”* ratio (dQx = Qx_INVIVO_/Qx_INVITRO_) was calculated for each STM barcoded strain, therefore normalizing the *in vivo* data to the *in vitro* growth of each mutant strain. Moreover, to obtain the FB score for each strain [S = log2 (dQx/dQ_STM6_)], each dQx ratio was normalized to the dQ_STM6_ of the pool to normalize the results of all pools. The STM6 barcode was present in isogenic wild-type strains present in all the pools as a positive control.

### RNA Extraction and Reverse Transcription qPCR (RT-qPCR)

In order to verify the expression of *ZCF6* and *MBF1*, RNA was extracted from wild-type *C. albicans* cells (strain SC5314) in exponential growth phase from *in vitro* cultures. It was also extracted from infected mouse kidneys 48 h pi and from infected *G. mellonella* 24 h pi. *C. albicans in vitro* cultured cells were washed twice in PBS and then flash-frozen with liquid nitrogen (N_2_). Mice kidneys were collected and directly placed in RNAlater solution, then transferred to a mortar and flash-frozen with N_2_. Larvae were directly placed in a mortar and sacrificed by flash-freezing with N_2_. All samples were then processed as follows: samples were ground until reduced to thin powder. Approximately 100 mg of this powder was immediately suspended in 600 μl of Trizol^®^ (Life Technologies), 300 μl of phenol-chloroform-isoamyl alcohol 25-24-1 (Sigma), 300 μl of RNA buffer (0.1 M Tris HCl pH 7.5, 0.1 M LiCl, 10 mM EDTA, 0.5% SDS) and the volume equivalent to 200 μl of acid-washed 0.5 mm-diameter glass beads in a screw-capped RNase-free 2 ml tube. Cells were disrupted in a FastPrep 24 machine at 6.5 m/s for 15 s. After 5 min of centrifugation at 4∘C at 13000 *g*, supernatants were transferred to a new RNase-free 1.5 ml microcentrifuge tube and one volume of phenol-chloroform-isoamyl alcohol 25-24-1 was added and vortexed for 10 s. The centrifugation was repeated and the supernatant was transferred to a new RNase-free 1.5 ml microcentrifuge tube. RNA was precipitated with one volume of EtOH 100% and the solution was loaded into columns of the DirectZol^TM^ RNA MiniPrep kit (Zymo Research), thereafter following the instructions of the manufacturer. A DNase treatment was included in the kit and carried out in-column. Two μl of RNasin^®^ Plus RNase Inhibitor (Promega) were added to the final RNA extracts and these were kept at -80∘C until further use. For RT-qPCR, one microgram of RNA (determined by NanoDrop 1000 Spectrophotometer, Thermo Fisher Scientific) was reverse transcribed using random hexamers as a priming method (Transcriptor High Fidelity cDNA Synthesis Kit, Roche). Subsequent RT-qPCR reactions were performed with 0.2 μM of each primer and 0.2 μM of probe for genes *ACT1*, *MBF1,* and *ZCF6* (see Supplementary Table S1), and iTAQ Supermix with ROX (BioRad, Reinach, Switzerland) according to the manufacturer’s instructions, using StepOnePlus^TM^ Real Time PCR System (Life Technologies). The expression level of *ACT1* was used for normalization, and fold change values were calculated for *MBF1* and *ZCF6 in vivo* versus *in vitro*. Technical duplicates were included in each reaction, and all reactions were repeated twice on biological duplicates.

### *G. mellonella* Infection Model

*Galleria mellonella* larvae were purchased from Bait Express GmbH (Basel, Switzerland). Upon arrival, the larvae were stored with wood shavings at 12∘C in the dark and subsequently used within a maximum of 2 weeks. Larvae with a weight ranging from 300 to 400 mg were used for the experiments.

Only single strain infections were performed in *G. mellonella*. To determine the CFUs, groups of 5–10 larvae were used. Each larva was injected through the last left proleg with 40 μl of a cell suspension containing 2.5 × 10^6^ cells/ml using a Myjector U-100 insulin syringe (Terumo Europe). A control group, injected with 40 μl of sterile PBS, and a group of non-injected larvae were included. To verify that the injected volume did not negatively affect the larvae, we investigated the larvae survival following five repetitive injections of 40 μl PBS over 2 days compared with non-injected larvae. No negative effect was observed (data not shown). We also compared the survival of larvae injected once with 40 μl of wild-type *C. albicans* using a Myjector U-100 insulin syringe versus larvae injected with 10 μl of the same strain using a Hamilton syringe (same number of cells injected). No differences were observed (data not shown). The larvae were maintained at 30∘C in the dark, without food, and sacrificed at 24 h post-infection. Subsequently, the fungal load of each individual larva was determined. Briefly, each larva was homogenized in screw-cap tubes containing a metal bead [stainless steel, 7 mm, (VWR International)] with three rounds of shaking for 10 s at 6.5 m/s using a MP FastPrep^®^-24 (MP Biomedicals). The homogenate was immediately resuspended in 6 ml PBS, and two 10-fold serial dilutions in PBS were prepared. From each of the three dilutions, 100 μl were plated onto YEPD-chloramphenicol (50 μg/ml) plates and incubated at 30∘C for 48 h. The CFU were enumerated and expressed for each larva as a percentage of the mean of the fungal load of the group infected with the wild-type strain in the same experiment. Each mutant was tested (i) twice using a minimum of five larvae (both experiments were merged in Experiment 1), (ii) once using a minimum of 10 larvae (Experiment 2), or (iii) both (see File S1). Statistical analyses were performed using a ROUT analysis (Motulsky and Brown, 2006) to remove outliers, followed by the Mann–Whitney test to assess CFU differences relative to the wild-type strain. For survival experiments, groups of 12–15 larvae were used, and each mutant was tested three times. The larvae were inoculated with 40 μl of *C. albicans* cell suspension containing 1.25 × 10^7^ cells/ml. A control group, injected with 40 μl of sterile PBS, and a non-injected larvae group were included. The larvae were maintained at 30∘C in the dark, without food, and monitored twice daily. The post-infection day of natural death was recorded for each larva. The larvae were considered dead when no movement and no response to a physical stimulus applied with tweezers were observed. The survival experiments were terminated at 15–21 days after infection. Even if this time lapse is usually sufficient for the larvae to initiate metamorphosis, infection clearly seemed to block it and therefore this process was not observed during survival experiments. The statistical analyses of the survival data were performed using the log-rank (Mantel–Cox) test.

### *In Vitro* Phenotypic Tests

Fifty-two different *in vitro* growth conditions, mimicking the conditions relevant for systemic infection of the host, were used to determine the phenotype of each mutant strain compared with the wild-type parental strain. The yeast cultures were grown overnight in liquid YEPD and diluted to a concentration of 2.5 × 10^7^ cells/ml. Five 10-fold serial dilutions were prepared at a final concentration of 2.5 × 10^2^ cells/ml. Four microliters of each dilution were spotted onto YEPD-based plates and incubated for 24–72 h at 35∘C unless otherwise specified. First, heat and cold susceptibilities were tested through incubation at 42 or 16∘C compared with the reference condition at 35∘C. The strains were also grown in the presence of 5% CO_2_. The sensibility to alkaline (pH 8.5 or pH 9.3, achieved through the addition of HEPES 1 M pH 8.5 and NaOH 10 N, respectively) or acidic pH (pH 3.35 or pH 2, achieved through the addition of HEPES 1 M pH 1.9 and HCl 10 N, respectively) was also verified. The use of alternative carbon sources was assessed by growth on minimal medium (YNB) plates supplemented with 2% glucose, 2% galactose, 2% sorbose, 2% raffinose, 2% maltose, 1% ethanol, 2% citrate, 0.1% acetic acid, 0.1% lactic acid, 0.1% maleic acid, 1 mM sorbic acid, 2% glycerol, 0.1% sodium acetate, or 2% sorbitol. Growth in the presence of cell wall stress agents was determined after spotting the strains onto YEPD plates supplemented with calcofluor white (100 μg/ml), Congo red (100 μg/ml) or 0.02% sodium dodecyl sulfate (SDS). Susceptibility to oxidative stress was tested using YEPD medium containing 5, 10, or 20 mM H_2_O_2_. Resistance to salt stress was evaluated using YEPD medium supplemented with 1 M NaCl, 1 mM CuSO_4_, 400 mM CaCl_2_, 400 mM MgCl_2_, 200 mM LiCl, or 10 mM MnCl_2_. The growth of the mutated strains was also evaluated in YNB minimal medium without iron and calcium, or in the presence of the iron chelator bathophenanthroline di-sulfonic acid (BPS; YEPD containing 100 μM BPS). Susceptibility to several drugs was also determined after supplementing YEPD medium with fluconazole (10 or 40 μg/ml), fluphenazine (50 μg/ml), 4-nitroquinoline 1-oxide (4NQO; 10 μg/ml), caspofungin (0.1 μg/ml), amphotericin B (1.5 μg/ml), phloxine B (5 mM), rapamycin (5 mM), caffeine (2.5 mM), estrogen (20 μg/ml), benomyl (50 μg/ml), terbinafine (20 μg/ml), or flucytosine (5-FC; 4 μg/ml; this last drug was added to SD agar plates). Moreover, the filamentation phenotype was determined after plating each mutant onto YEPD agar plates supplemented with 10% fetal calf serum (FCS).

## Results

### *In Vivo* Screening of the TF Mutant Collection

To identify the TFs important for *C. albicans* invasion and host infection, we examined a collection of 234 mutants of putative TF genes using an *in vivo* model. This collection includes 77 TF mutants with a Zn_2_Cys_6_ cluster motif reported in a first analysis (Vandeputte et al., 2011) and another set (157 TF mutants) analyzed in the present work. In both studies, the mutants were tested in a mouse model of systemic infection through tail vein injection, and FBs were determined in the kidneys at three dpi. To reduce the number of animals needed, the mutants were tested in pools of 10 strains, including eight mutant strains, one wild-type strain and the calcineurin mutant strain (*cmp1*Δ/Δ) as an avirulent control. Each pool was injected into at least three mice (see File S1 and Vandeputte et al., 2011). We introduced a barcode to distinguish between the different strains. Thus, the relative number of cells in the kidneys could be easily quantified using qPCR based on the copy number of each barcode, as previously described (Vandeputte et al., 2011). A colonization score FB was determined for specific mutants relative to the wild-type strain (calculation detailed in Materials and Methods) to estimate the fitness of the isolates. Next, to determine whether mutants had FB scores significantly different from wild-type, a threshold of significance had to be determined. For this purpose, we barcoded the wild-type strains using 10 different tags, obtaining 10 different wild-type strains carrying a distinct barcode. Similar to the analysis of the TF mutants, a FB score was attributed to each wild-type barcoded strain relative to the strain carrying the STM6 barcode. In our first study (Vandeputte et al., 2011), we considered that strains with a score above or below that of the wild-type (mean ± 2 SD) had a FB score significantly different from that of the wild-type. However, when we determined in this study the efficacy of the qPCR targeting the 10 different barcodes, it ranged from 61.6 to 94.1% (Supplementary Table S4), thus introducing a bias in the analysis. In the present work, a bias correction was introduced in the analysis of all tested mutants as well as for those examined in our previous study (Vandeputte et al., 2011). We excluded the results obtained when the qPCR efficacy was below 80%, thereby excluding mutants bearing the tag STM224. Next, we used Mann–Whitney analyses to compare the scores of each mutant strain with those of the wild-type strain carrying the same barcode (File S1) to circumvent the qPCR bias. Mutants with less than three calculated scores were considered non-detected and were not included in the statistical analysis. Finally, with the obtained FB scores that were significant (47 mutants, *p* ≤ 0.05), individual mutants were ranked as low- or high- FB mutants. The final results are presented in Supplementary Figure S1 and **Tables 1A,B**. Twenty-six mutants exhibited low FB scores (*p* ≤ 0.05; **Table 1A**) and among them six mutants (*RIM101, NOT5, RFX1, SEF1, HAP43,* and *SPT3*) had been previously described as important for virulence (Davis et al., 2000; Laprade et al., 2002; Cheng et al., 2003; Hao et al., 2009; Hsu et al., 2011; Vandeputte et al., 2011). We also identified 21 high FB mutants (*p* ≤ 0.05) and among them two mutants (*UPC2* and *CTF1*) were previously reported as critical for virulence (Ramirez and Lorenz, 2009; Lohberger et al., 2014). Ramirez and Lorenz (2009) and Lohberger et al. (2014) reported that the *CTF1* and *UPC2* mutants, respectively, were less virulent than the wild-type, and this result was not consistent with the results obtained in the present study. Notably, the *CTF1* and *UPC2* mutants in Ramirez and Lorenz (2009) and Lohberger et al. (2014) were null mutants constructed in different genetic backgrounds and tested in single strain infections. In addition, 19 mutants could not be detected from kidneys of animals infected with pools (**Table 1B**). From these non-detected mutants, seven were previously reported to display decreased virulence in mice (Lo et al., 1997; Braun et al., 2000; Singh et al., 2004; Dagley et al., 2011; Yan et al., 2014). The remaining 12 non-detected mutants, not yet annotated, represent interesting candidates for further study (**Table 1B**).

**Table 1A:** **The results of mice pool infection experiments**.

FB scores significantly different from the wild-type strain (*n* = 47)^a^											
Low FB mutants (*n* = 26)						High FB mutants (*n*= 21)					
Non-annotated (*n* = **10**)^**b**^		Annotated with no reference to virulence **(***n* = **10)**^**b**^		Annotated with a role in virulence (*n* = **6)**^**c**^		Non-annotated (*n* = 9)^**b**^		Annotated with no reference to virulence (*n* = **10)**^**b**^		Annotated with a role in virulence (*n* = **2)**^**c**^	
**Mutated ORF^**d**^**	**Gene name**	**Mutated ORF**	**Gene name**	**Mutated ORF**	**Gene name**	**Mutated ORF**	**Gene name**	**Mutated ORF**	**Gene name**	**Mutated ORF**	**Gene name**
orf19.1497	*ZCF6*	orf19.4998	*ROB1*	orf19.7247	*RIM101*	orf19.2647	*ZCF14*	orf19.6038	*UGA32*	orf19.1499	*CTF1*
orf19.2646	*ZCF13*	orf19.2331	*ADA2*	orf19.5107	*NOT5*	orf19.4649	*ZCF27*	orf19.4288	*CTA7*	orf19.391	*UPC2*
orf19.1565	NA^e^	orf19.1693	*CAS4*	orf19.3865	*RFX1*	orf19.5924	*ZCF31*	orf19.3986	*PPR1*		
orf19.5552	NA	orf19.4961	*STP2*	orf19.3753	*SEF1*	orf19.7583	*ZCF39*	orf19.1543	*OPI1*		
orf19.2963	NA	orf19.7017	*YOX1*	orf19.681	*HAP43*	orf19.7547	NA	orf19.4545	*SWI4*		
orf19.3928	NA	orf19.4752	*MSN4*	orf19.7622	*SPT3*	orf19.1178	NA	orf19.4000	*GRF10*		
orf19.6713	NA	orf19.4318	*MIG1*			orf19.2458	NA	orf19.4662	*RLM1*		
orf19.2399	NA	orf19.1623	*CAP1*			orf19.259	NA	orf19.2087	*SAS2*		
orf19.6850	NA	orf19.1826	*MDM34*			orf19.5617	NA	orf19.6781	*ZFU2*		
orf19.5651	NA	orf19.723	*BCR1*					orf19.6904	*GCN3*		

*^a^Mutants listed with kidney fungal burden (FB) scores with significant statistical differences from the wild-type strain carrying the same STM-tag (p≤ 0.05) in mouse pool experiments were divided as low- or high- FB mutants; ^b^Groups of mutants tested in G. mellonella; ^c^Mutants were further divided according to the annotation in CGD (Candida Genome Database); ^d^ORF names according to the C. albicans genome assembly 21. The corresponding assembly 22 names are indicated in File S1; ^e^NA, not available*.

**Table 1B:** Results of mice pool infection experiments.

Mutants non-detected (*n* = 19)^a^						Mutants tested in mice but excluded due inefficient qPCR (*n* = 26)					
Non-annotated (*n* = 6)^b^		Annotated with no reference to virulence (*n* = 6)^b^		Annotated with a role in virulence (*n* = 7)^c^		Non-annotated (*n* = 8)		Annotated (*n* = 18)^c^	
Mutated ORF^d^	Gene name	Mutated ORF	Gene name	Mutated ORF	Gene name	Mutated ORF	Gene name	Mutated ORF	Gene name	Mutated ORF	Gene name
orf19.6227	NA^e^	orf19.3294	*MBF1*	orf19.5734	*POP2*	orf19.1576	NA	orf19.1253	*PHO4*	orf19.6124	*ACE2*
orf19.1697	NA	orf19.5992	*WOR2*	orf19.610	*EFG1*	orf19.2612	NA	orf19.3018	*SPP1*	orf19.6817	*FCR1*
orf19.6506	NA	orf19.5855	*MBP1*	orf19.6109	*TUP1*	orf19.3833	NA	orf19.4433	*CPH1*	orf19.6985	*TEA1*
orf19.5666	NA	orf19.5871	*SNF5*	orf19.971	*SKN7*	orf19.719	NA	orf19.4941	*TYE7*	orf19.2623	*ECM22*
orf19.5910	NA	orf19.6121	*MNL1*	orf19.2315	*RTG3*	orf19.6861	NA	orf19.5580	*TEL1*	orf19.3012	*ARO80*
orf19.5953	NA	orf19.6011	*SIN3*	orf19.5908	*TEC1*	orf19.4145	*ZCF20*	orf19.59	*REI1*	orf19.5729	*FGR17*
				orf19.4670	*CAS5*	orf19.4524	*ZCF24*	orf19.6849	*ELC1*	orf19.7317	*UGA33*
						orf19.4767	*ZCF28*	orf19.798	*TAF14*	orf19.1759	PHO23
								orf19.2356	*CRZ2*		
								orf19.5133	*RLS17*		

*^a^Mutants not detected in mice pool experiments for which no score could be calculated; ^b^Mutants tested in G. mellonella; ^c^Mutants were further divided according to the annotation in CGD (Candida Genome Database); ^d^ORF names according to the C. albicans genome assembly 21. The corresponding assembly 22 names are indicated in File S1; ^e^NA, not available*.

To estimate the validity of the approach used herein, we compared the results in the present study with annotations of null mutants examined using so-called “classic genetics” (i.e., individual testing in mice) and available in the Candida Genome Database^2^. For this analysis, we considered the non-detected strains and the low FB phenotype strains in the same group, reasoning that the lack of detection of the mutants reflected an extremely low FB. We observed that 62.5% of the mutants examined here showed a matching phenotype with respect to virulence when compared to previous annotations (File S1-Venn Diagram and Supplementary Table S5). In this group, we identified genes critical for pH (*RIM101*) or iron homeostasis (*SEF1* and *HAP43*) and yeast-hypha switching (*EFG1, TUP1, NOT5,* and *SPT3*). The validation of our assays is therefore supported by these results.

Recent population genetics analyses have shown that strain communities, such as mutant pools, experience a phenomenon called “clonal interference,” which can be considered as a “pool effect” (see Discussion; Vandeputte et al., 2011), which might bias the phenotype of a given mutant. Therefore, we sought to confirm the low- and high FB mutant phenotypes obtained in the pool infection by performing single strain infections. For this purpose, mutants with annotations referring to altered virulence were excluded (8 out of 47 mutants), thus yielding 39 interesting mutants (**Table 1A**). We also assessed the phenotypes of 12 non-detected mutants with no previous annotations relative to virulence in single strain infections (**Table 1B**). Taken together, 51 mutant strains (39 with a significant FB score but not yet annotated and 12 not detected in pool infections of mice) were selected for testing through single strain infections.

### Screening Candidate TF Mutants in the Alternative *G. mellonella* Model

**FIGURE 1:**
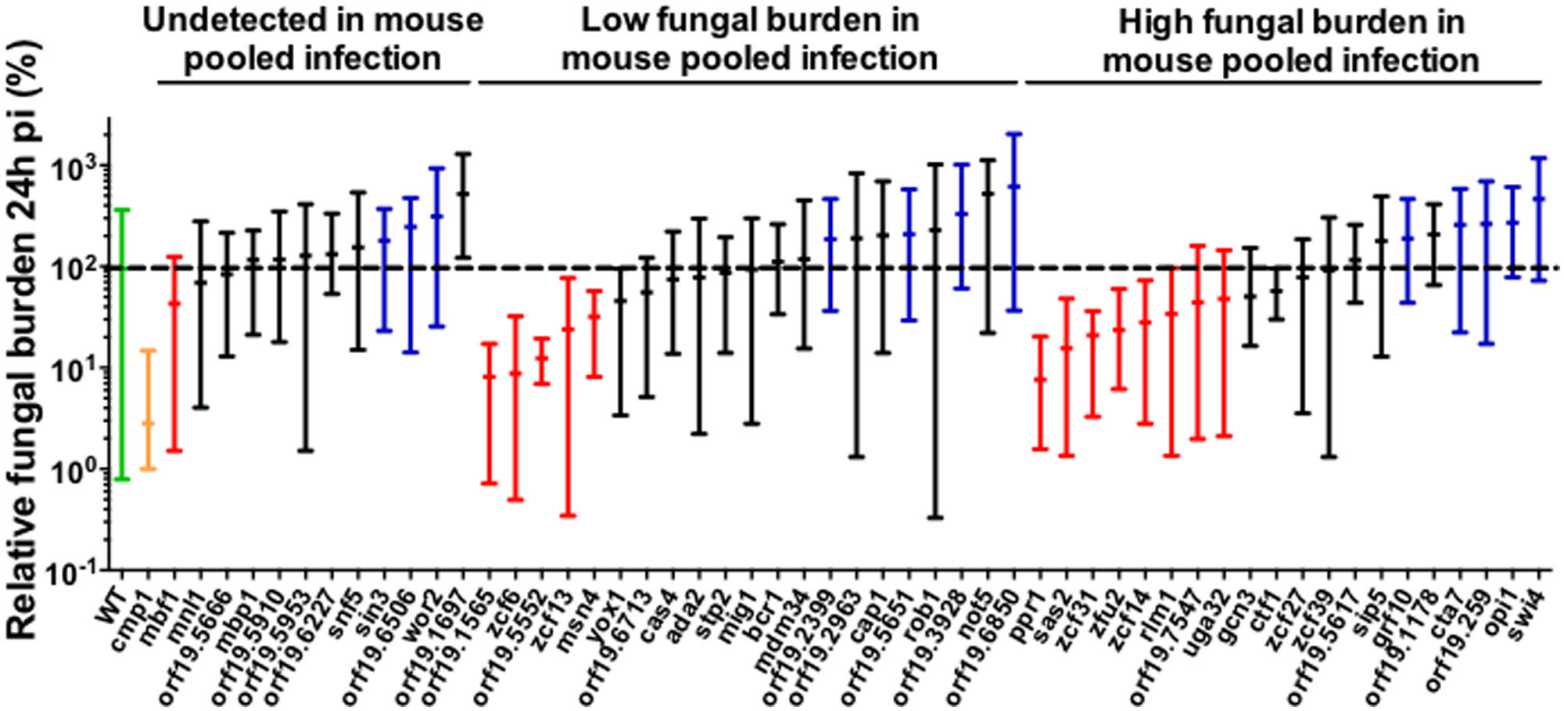
**Fungal burden (FB) of BCY mutants in *G. mellonella* single strain infections**. For each individual larva, the FB is expressed as a percentage relative to the mean of the wild-type group in the same experiment. This normalization enables comparisons between different experiments. All *Galleria mellonella* experiments are pooled in this graph. The results are expressed as the mean of the group and the minimum and maximum values. The results are organized according to the phenotype observed in mice pool infections. The green bar corresponds to the wild-type strain. The yellow bar corresponds to the *cmp1*Δ/Δ mutant (avirulent control). The red bars correspond to low FB mutants significantly different from wild-type in *G. mellonella*. The blue bars correspond to high FB mutants significantly different from wild-type in *G. mellonella*. The black bars indicate mutants not significantly different from the wild-type. The dotted line indicates the level of the mean of the wild-type strain set at 100%. Each mutant was tested at least twice using a minimum of five larvae each time or once using a minimum of 10 larvae. Statistical analyses were performed using a ROUT analysis to remove outliers (Motulsky and Brown, 2006), followed by a Mann–Whitney test to assess CFU differences relative to the wild-type strain.

A high number of animals are required to individually examine 51 mutants in mice, which is not ethically suitable. Therefore, we used the larvae of the greater wax moth, *G. mellonella*, to perform single strain infections. *G. mellonella* is a model organism used in recent studies to examine fungal virulence and antifungal drug efficacy. This *in vivo* model of systemic infection provides results closely correlating with those obtained using the standard mouse model (reviewed in Coste and Amorim-Vaz, 2015). Most studies perform survival tests using individual strains in this insect model. In the present study, we assessed the larvae FB based on CFU counting to compare them with the results obtained from the initial screening using the mouse model. To our knowledge, this comparison has not been previously attempted at such a large scale. First, we performed a preliminary experiment using a wild-type strain to determine the optimal inoculum for injection and the optimal lapse time before CFU counting. The results are shown in Supplementary Figure S2. We determined that 24 h post-infection was a better time-point than 48 h to obtain a clear dose-response (from 10^3^ to 10^6^ cells per larva) in which most of the larvae survived. We used 10^5^ cells per larva as inoculum to remain within the linear range of the dose-response curve. Next, we screened the 51 selected mutant strains, and the results are shown in **Figure 1**. We used the calcineurin null mutant (*cmp1*Δ/Δ) as an avirulent control in *G. mellonella.* The CFU counts determined from *cmp1*Δ/Δ strain infected larvae revealed significantly lower FBs than those obtained with the wild-type strain, thereby validating this approach. Using the mean FB of the wild-type strain (included in each experiment and set at 100%) as a reference, low- and high- FB mutants were considered below and above the 100% threshold, respectively, considering a *p* ≤ 0.05 (Mann–Whitney test). Interestingly, in this assay, high variability was observed with the wild-type strain. In the present study, the difference in CFU counting in *G. mellonella* larvae was as high as 100-fold for the same mutant (**Figure 1**). After organizing the results according to the phenotype observed in the mouse model of pool infections, we observed that several low FB mutants in mice switched phenotype in *G. mellonella* (change from low- to high- FB phenotypes) or lost phenotype in this host (no difference between wild-type and mutant strains). The same pattern was observed for mutants with high FB phenotype in mice. Thus, in contrast with previous reports, we observed significant discrepancies between the results obtained using these two models of infection when analyzing FBs in target organs in mice and *G. mellonella*, with only 21.5% of matching results between the two sets of experiments (File S1-Venn Diagram and Supplementary Table S5). This value could be increased to 30.7% using another statistical approach, in which multiple *t*-tests were corrected using FDR (0.05; Supplementary Table S5). However, increasing the stringency of the statistics eliminates several virulence genes, such as *SEF1* (File S1). To maintain as many virulence gene candidates as possible, we selected in the present study the less stringent Mann–Whitney and non-FDR-corrected analysis. One important consideration is that in addition to the two different model organisms, the mode of infection also changed as the mutants were tested in pools in mice but individually in *G. mellonella*. Hence, these two sets of experiments confounded two variables, including the type of host and the type of inoculum (single strain versus pool).

### Comparison of Single Strain Infections of *G. mellonella* and Mice Injected with TF Mutant Strains

Among the 51 selected mutants tested in *G. mellonella,* we used 18 mutants to perform single strain infections in the mouse model to facilitate a direct comparison of the two model organisms without the influence of the “pool effect”. This approach may also facilitate the comparison of the two modes of infection (pooled or single strain infections), irrespective of the host. For this comparison, we used mutants with (i) the same FB phenotypes in the *G. mellonella* single strain infection and mouse pool infection (i.e., *CTA7*, *ZCF6*, *OPI1*, *ORF19.1565*, *ORF19.6713*, *GRF10*, and *SWI4*), (ii) opposite FB phenotypes (*ZCF14*, *ZCF31*, *ZFU2*, *RLM1*, *ORF19.2399*, and *ORF19.3928*) in the two animal models, (iii) a wild-type phenotype in *G. mellonella*, but significantly altered in the mouse pooled infections (*ZCF39*, *ORF19.1178*, *MDM34*, and *ORF19.5617*), and (iv) non-detected in mice, but a pronounced low FB phenotype in *G. mellonella* (*MBF1*). This 18 selected mutants were tested twice (Experiment 1 and Experiment 2, see file S1) in single strain infection in at least five mice each time. We also added to these results the data previously obtained with the *ZCF13* mutant in the mouse single strain infection (Vandeputte et al., 2011) since this mutant has potential interest.

**FIGURE 2:**
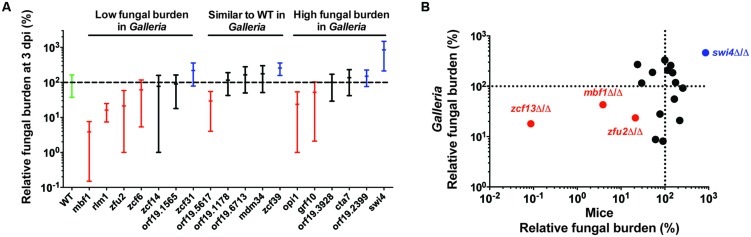
**Comparison of the results obtained using 19 mutant strains in two different *in vivo* models, including *G. mellonella* and mice**. **(A)** Fungal burden of BCY mutants in mice single strain infections. For each individual mouse, the FB in the kidneys is expressed as a percentage relative to the mean of the wild-type group in the same experiment (set at 100% and indicated as a dotted line). All experiments are pooled in this graph. Only the results from mutants tested at least twice are presented here. The results are organized according to the phenotype observed in *G. mellonella* single strain infections. The green bar corresponds to the wild-type strain. The red bars correspond to statistically low FB mutants in mouse single strain infections. The blue bars correspond to high FB mutants significantly different from the wild-type in mouse single strain infections. The black bars indicate no significant difference from the wild-type. Each mutant was tested twice using five mice each time. The statistical analyses were performed using a ROUT analysis to remove outliers, followed by a Mann–Whitney test to assess CFU differences relative to the wild-type strain. The horizontal bars indicate the mean and minimum–maximum of FB for each mutant strain. **(B)** Correlation between results obtained with *G. mellonella* (24 h pi) and mice (three dpi). Red and blue data points correspond to mutants with the strongest alterations in host systemic infectivity (low- and high- FB mutants, respectively). The data for the *zcf13* mutant were obtained from Vandeputte et al. (2011). Spearman’s rank correlation coefficient: *r* = 0.30, *p* = 0.2. Excluding colored data points, *r* = -0.17, *p* = 0.56. The dotted lines indicate the level of the mean of the wild-type strain set at 100% in each model.

The results obtained in mouse model of single strain infections using these 18 strains are presented in **Figure 2A**. The results are expressed as a percentage of the mean FB for the wild-type of each experiment. Comparison of these results with those obtained in *G. mellonella* experiments, including FB values of the previously tested *zcf13* mutant strain (Vandeputte et al., 2011), provide information on the degree of the host effect. Ten mutants (∼50%) showed matching phenotypes in the two models: five low FB mutant strains (*rlm1-, zcf6-, zcf13-, mbf1-,* and *zfu2* mutant strains), two high FB mutant strains (*swi4-* and *orf19.2399* mutant strains) and three wild-type strains (*orf19.1178-, orf19.6713-,* and *mdm34* strains). The remaining nine mutants showed conflicting phenotypes in the two model hosts. These results suggest that the phenotypes observed for ∼50% of the strains were dependent on the host (Supplementary Table S5 and File S1-Venn Diagram).

The correlation between the mean FBs for the 19 analyzed *G. mellonella* and mouse mutants (as a percentage relative to the wild-type) are shown in **Figure 2B**, including the *ZCF13* mutant examined in mice in a previous study (Vandeputte et al., 2011). The apparent correlation primarily reflected the edges of the plot, in which the fungal load of the infected group containing three low- mutants (*ZFU2*, *ZCF13*, and *MBF1*) and one high FB mutant (*SWI4*) is highlighted among the middle aggregate data. Considering these observations, *G. mellonella* is an interesting *in vivo* model for the detection of mutants in which infectivity is strongly affected. Indeed, these four mutants displayed an FB at least five times higher or lower than the FB of the wild-type strain (**Figure 2B**).

The comparison of the results obtained in single strain with pool infections in mice, including FB values of the previously tested *zcf13* mutant strain (Vandeputte et al., 2011), was used to estimate the degree of the “pool effect”. For the mutants examined in the present study, we observed matching phenotypes for only 33.4% of the strains with *p* < 0.05 and even to 50% when applying FDR (0.05; **Table 1**, **Figure 2**, Supplementary Table S5 and File S1-Venn Diagram). These results indicate that around 50% of the phenotypes observed in the mice pool infection experiments were influenced by the pool effect.

We reasoned that the most interesting mutants were those for which infectivity was independent of the host or the pool effect. Consequently, we further investigated mutant strains with low FB phenotypes in the three tested infection models, namely the *zcf6-*, *zcf13-,* and *mbf1* mutant strains.

### Infection Assays with Independent Mutants

Notably, the selected mutants were constructed using two different strategies: UAU transposition (Davis et al., 2002) in BCY130 (mutant for *ZCF6*) and BCY152 (mutant for *ZCF13*), and 100-mer extension from pFA plasmids (Gola et al., 2003) in BCY431 (mutant for *MBF1*). Therefore, these mutants contain partial gene deletions and/or exhibit the ectopic expression of auxotrophic markers. Importantly, the ectopic expression of *URA3* might influence the differences observed in the virulence phenotype of *C. albicans* strains (Brand et al., 2004). To exclude the possibility that the phenotypes observed in the present study reflected artifacts in the *C. albicans* genome as a consequence of these strategies, we examined a set of independent mutants of the three candidate genes described above (*ZCF6*, *ZCF13,* and *MBF1*) using the *SAT1* flipper cassette (Reuss et al., 2004) and SC5314 as the parental strain. We also constructed revertant strains from each mutant through the re-insertion of the wild-type alleles at one deleted locus. None of these strains displayed growth defects *in vitro* (Supplementary Figure S3).

**FIGURE 3:**
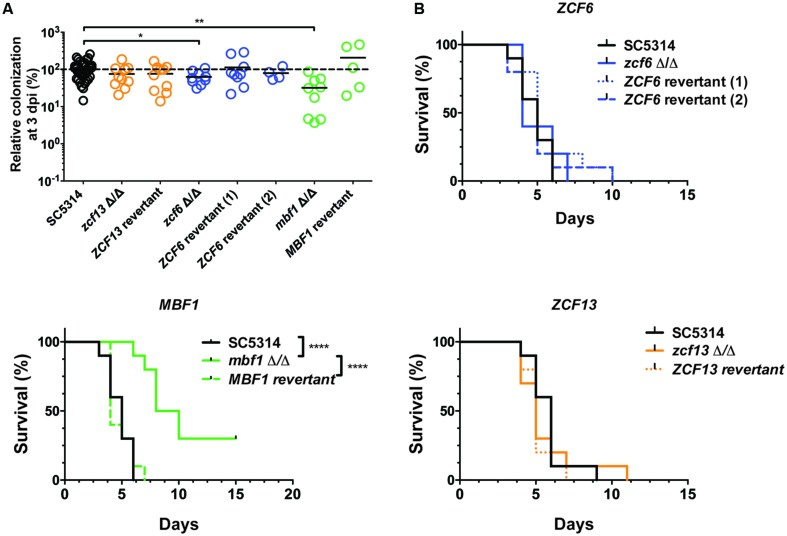
**Testing an independent set of mutants in mice**. **(A)** Fungal burden of full ORF, marker-free *C. albicans* deletion mutants in mice single strain infections. For each individual mouse, the FB in the kidneys is expressed as a percentage relative to the mean of the wild-type group in the same experiment. All experiments are pooled in this graph. The mean value of each group is indicated with a black bar. The dotted line indicates the level of the mean of the wild-type strain set at 100%. Each mutant was tested twice using five mice each time (except for *ZCF6* revertant 2 and *MBF1* revertant). Statistical analyses were performed using a Mann–Whitney test to assess CFU differences relative to the wild-type strain. The stars indicate the level of statistical significance: ^∗^*p*< 0.05, ^∗∗^*p* < 0.01. **(B)** Survival curves in mice. The mice were injected with 5 × 10^5^ cells of the corresponding strain and monitored daily. These survival curves represent at least two independent experiments. Groups of 10 mice were used each time. ^∗∗∗∗^*p*< 0.0001, log-rank test.

**FIGURE 4:**
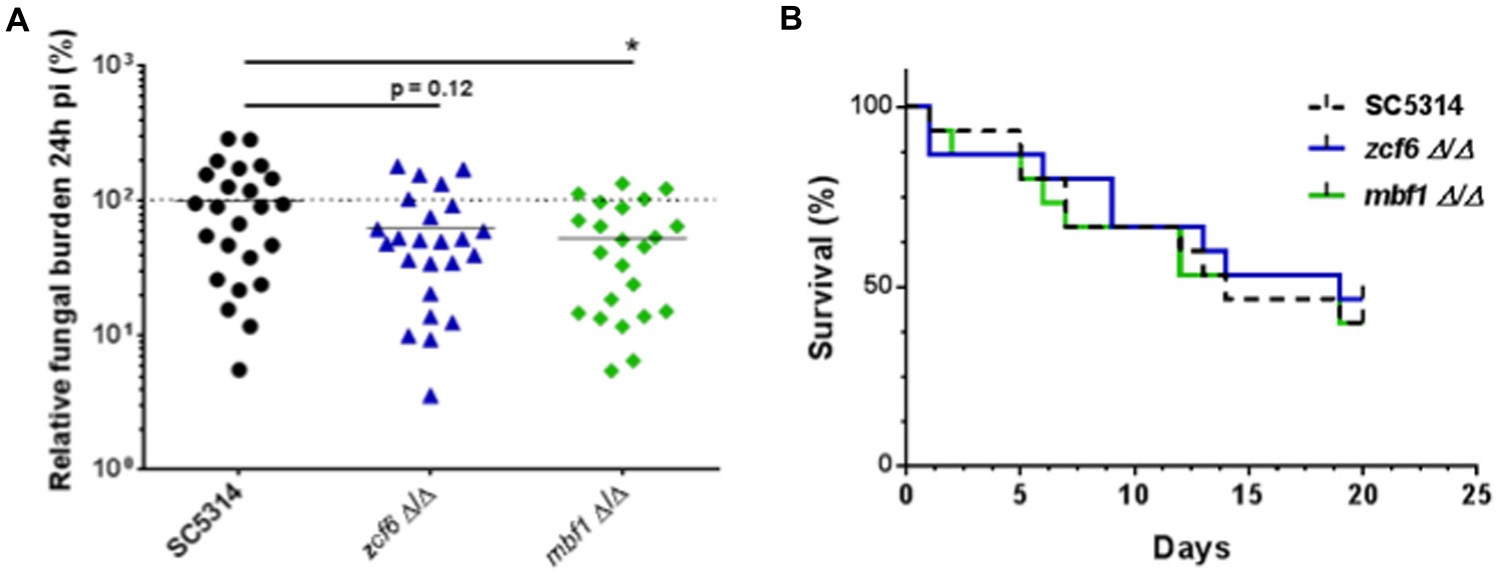
**Testing an independent set of mutants in *G. mellonella*. **(A)** Fungal burden of full ORF**, marker-free deletion mutants in *G. mellonella* single strain infections. For each individual larva, the FB is expressed as a percentage relative to the mean of the wild-type group in the same experiment. All experiments are pooled in this graph. The mean of each group is indicated with a black bar. The dotted line indicates the level of the mean of the wild-type strain set at 100%. Each mutant was tested three times using at least six larvae each time. Statistical analyses were performed using a ROUT analysis to remove outliers (Motulsky and Brown, 2006), followed by a Mann–Whitney test to assess CFU differences relative to the wild-type strain. The star indicates the level of statistical significance: ^∗^*p* < 0.05. **(B)** Survival curves in *G. mellonella*. Larvae were injected with 5 × 10^5^ cells of the corresponding strain and monitored twice daily. These survival curves represent three independent experiments. Groups of at least 10 larvae were used each time. The log-rank test was performed, and no significant differences were observed.

The independent mutants for *ZCF6, ZCF13,* and *MBF1* were subsequently tested using a mouse model of systemic infection. The FB in the kidneys was measured at 3 dpi (**Figure 3A**), and survival curves were obtained for each mutant (**Figure 3B**). In terms of systemic infection in the mouse (kidney FBs), mutants for *ZCF6* and *MBF1* confirmed the previously observed low FB phenotype (*mbf1*Δ/Δ: *p* = 0.0002, *zcf6*Δ/Δ: *p* = 0.033). Revertant strains, in which the wild-type allele was reintroduced, were also tested, revealing the same level of kidney FBs as observed in the parental wild-type strain. The full ORF, marker-free deletion mutant for *ZCF13* showed a phenotype similar to that of the wild-type, therefore the low FB phenotype in BCY152, obtained through UAU transposition, was not observed with the independent deletion mutant. In parallel, the three mutant strains were examined in survival experiments. Only the *MBF1* mutant showed attenuated virulence, with a median survival time of 9 days compared with 5 days for the wild-type and 4 days for the revertant strain (*p* < 0.0001 in Log-rank test; **Figure 3B**). The *MBF1* mutant also displayed a significant low FB phenotype in *G. mellonella* (*p* < 0.05, **Figure 4A**). In contrast, no significant differences in larvae fungal loads were observed for the *ZCF6* mutant compared with wild-type (*p* = 0.12; **Figure 4A**). Nevertheless, this mutant showed a clear tendency for lower infection of the larvae than the wild-type, and this tendency was reproducible across two different experiments (mean Experiment 1 = 70.2%, mean Experiment 2 = 56.9% of the wild-type mean). Interestingly, the variability observed in *G. mellonella* was consistently higher than that observed in the mouse model (**Figures 3A and 4A**), thus masking statistically significant results (considering *p*-values ≤ 0.05 as threshold). These two mutants were further evaluated in survival experiments in *G. mellonella* (**Figure 4B**), and the results showed that none of the mutants exhibited a survival phenotype different from the wild-type.

### *In Vitro* Phenotypic Characterization

To identify the biological processes involving the mutated genes, we examined mutants that maintained a coherent phenotype throughout the course of the study (*MBF1* and *ZCF6*) and the corresponding revertant strains under 52 different *in vitro* conditions. This allowed assessing the susceptibility of the TF mutants to a variety of stresses (see Materials and Methods). Except for *mbf1*Δ/Δ, which showed slightly increased resistance to fluconazole, no phenotype could be observed for the two mutants under any of the conditions examined (Supplementary Figure S3). This suggests that these genes are specifically required for *in vivo* conditions.

### Expression of *MBF1* and *ZCF6 In Vitro* and *In Vivo*

Since no *in vitro* phenotypes were observed with the two tested mutants, it is possible that *MBF1* and *ZCF6* are not expressed *in vitro*. Thus their deletion would not yield any phenotype. We therefore verified their expression both *in vitro* and *in vivo*. However, expression of both genes could be measured *in vitro* and *in vivo* from mouse kidneys (48 h post-infection) or *G. mellonella* larvae (24 h post-infection; Supplementary Table S6). These results demonstrate that the absence of *in vitro* phenotypes for the *mbf1Δ/Δ* and *zcf6Δ/Δ* mutants is not due to lack of gene expression, but rather reflects the use of phenotypic tests that did not match the characteristics of these mutants.

## Discussion

In the present study, we identified two new genes, *MBF1* and *ZCF6*, necessary for the systemic propagation of *C. albicans* in the mammalian mouse model, but only one of these genes (*MBF1*) was defective in the insect *G. mellonella*. Simultaneously, we compared these two animal models of infection at a large scale. We consider *G. mellonella* as an interesting model for preliminary large-scale screenings to identify mutants with severe virulence defects *in vivo*.

### Screening of a *C. albicans* TF Mutant Collection

We examined here a collection of *C. albicans* putative TF mutants in an intravenous systemic mouse infection model using pools of 10 strains at a time (eight mutants and two controls). This strategy spares animals and resources for identifying new potentially interesting mutants. We observed that the phenotypes obtained in the mouse pool infection experiments were 62.5% similar to the phenotypes previously reported in studies using classic genetics experiments, i.e., experiments in which the strains were individually tested (see Supplementary Table S5). The differences for the remaining 37.5% of the mutants can be attributed to different reasons. First, the number of animals used in our screening experiments was low (3–6 animals/pool). Second, the annotations in CGD were obtained with mutants constructed using different methods and different genetic backgrounds. Third, these differences can be due to competition between strains and/or to the phenomenon of clonal interference. Experiments performed on populations of bacteria or yeasts have demonstrated that the success of a given mutant strain reflects not only the effect of its mutation itself but also the effect of the genetic background of the remaining population in the pool (Lang et al., 2013; Hammer et al., 2014; Wolter and Hoffman, 2014). Despite these drawbacks, we conclude here that pool infection experiments are still trustworthy to narrow down the numbers of candidate mutants.

Nevertheless, one of the problems when quantifying the relative distribution of barcoded mutants in pools by PCR approaches is that the PCR reactions are not equally efficient for each barcode. In the present work, we corrected for this bias. When re-evaluating data obtained in our previous study with this correction (Vandeputte et al., 2011), we observed some discrepancies in the ranking of high and low FB mutants. As an example, we considered all zinc cluster TF mutants tested in single strain infections and reported here or in separate studies (Chiranand et al., 2008; Ramirez and Lorenz, 2009; Chen et al., 2011; Vandeputte et al., 2011; Lohberger et al., 2014). We identified 19 zinc cluster mutants with annotations regarding virulence. Among them, 61% were assigned the same FB phenotype in the present study. In contrast, only 32% were detected in our previous analysis using a non-corrected approach for qPCR efficiency (Vandeputte et al., 2011). These results clearly emphasized the need to carefully analyze population distribution data obtained by PCR approaches. The use of universal primers for tag amplification coupled with deep sequencing technologies may be better suited for future population quantifications.

### *G. mellonella* as an Infection Model to Study *C. albicans* Virulence Factors

The *G. mellonella* model has been used by other groups, who reported excellent correlations between the results obtained with the insect model and those obtained with mice in survival experiments (Cotter et al., 2000; Brennan et al., 2002). These previous studies used mutants for genes with well-known and strong virulence defects, including *EFG1*, *CPH1, CEK1, CDC35* (Brennan et al., 2002) and *CMP1* (Bader et al., 2006). In the present study, we did not estimate survival rates, but rather measured the capacity of the mutants for systemic proliferation in the host. In our hands, however, matching results between mouse and *G. mellonella* single strain infections were observed in 50% of the cases (**Figure 2**). The correlation between these two groups of results primarily reflected the extreme phenotypes of the strains that were strongly different from the wild-type in mice (fivefold less or more kidney fungal loads than wild-type), also observed as low- or high FB mutants in *G. mellonella*, respectively. Thus, we concluded that this insect model is useful for identifying mutants which exhibit major infection defects in mice, potentially explaining why previous studies performed in *G. mellonella*, using *C. albicans* mutants with strong virulence defects in mice showed good virulence correlations between the two models. However, mutants with moderate (but statistically significant) alterations in the FB phenotype in mice can be predicted to exhibit large discrepancies between the two different hosts.

The difficulty to reach statistical significance using the insect model might be explained by the increased variability of the results. Comparing the 25–75 percentiles of the results observed in **Figures 1** and **2** for the wild-type, the variability observed using the insect model was around 1,5-fold higher than in mice (*G. mellonella* model: 33.1–154.1%; mouse model: 50–143.4%). This variability also leads to a lower reproducibility of the experiments than in mice. Comparison of the two rounds of experiments in *G. mellonella* (Experiment 1 and Experiment 2) showed only 45.2% matching phenotypes, in contrast to the 61.1% matching phenotypes obtained between the two rounds of experiments in mice (Supplementary Table S5). *G. mellonella* larvae were obtained from a commercial supplier not aimed for use in research, likely contributing to this variability. Larvae were not inbred (in contrast to mice) and the sanitary conditions, temperature, and nutrition conditions of the insect storage and transport are unknown. Another recent study in *Cryptococcus neoformans* reports similar variability problems using this model. The authors clearly showed that *G. mellonella* were obtained from different suppliers, and even different batches from the same supplier generated different results (Eisenman et al., 2014). To overcome this problem, *G. mellonella* cultures should be maintained in a standardized manner to facilitate the comparison of results between different laboratories. However, standardized culture maintenance would be a heavy burden for investigators in terms of time consumed and logistical arrangements.

### Assessing the Virulence of an Independent Set of Mutants

Subsequent to screening in two hosts, we selected for further analyses mutants for *ZCF6*, *ZCF13,* and *MBF1*. The *C. albicans* mutants in the collection examined in the present study were obtained using different strategies: UAU-Tn7 transposition (Davis et al., 2002), 100-mer extension from pFA plasmids (Gola et al., 2003), and 100-mer extension from UAU transposon (Davis et al., 2002) or URA3 blaster (Fonzi and Irwin, 1993). Notably, the ectopic expression of *URA3* was later shown to cause altered virulence in *C. albicans* (Brand et al., 2004). In addition, the use of the UAU cassette to generate homozygous deletion mutants can result in chromosome aneuploidy in *C. albicans* (Legrand et al., 2008). Moreover, gene disruption through transposons can result in the expression of truncated gene products with unknown effects on the cell (Lo et al., 2003). Thus, after selecting the three most interesting *C. albicans* mutant strains, we constructed independent, full ORF marker-free deletion mutants for the same genes using the *SAT1* flipper cassette. However, the *ZCF13* marker-free full ORF deletion strain did not reproduce the low FB phenotype obtained with the BCY152 strain (*ZCF13* obtained by UAU-Tn7 transposition). Interestingly, the revertant of BCY152, in which one copy of the wild-type gene was re-introduced, restored the wild-type FB phenotype in mouse kidneys (Vandeputte et al., 2011). Thus, it is clear that the reduced capacity of BCY152 for host systemic infection is caused by an insertional mutation in *ZCF13* (Vandeputte et al., 2011). Given that the complete deletion of this gene does not lead to the same phenotype, we hypothesized that the mutation (and not a deletion) in this gene is responsible for the observed phenotype. Particularly, the insertion of a Tn7 transposon could be responsible for the premature polyadenylation of the ORF, leading to the translation of a truncated protein in BCY152. In a previous study, Lo et al. (2003) designed a TAGKO (transposon arrayed gene knockout) mutant in filamentous fungi and demonstrated that the Tn7 insertion lead to the premature polyadenylation of TAGKO transcripts, thereby leading to the translation of truncated proteins in fungal mutants (Lo et al., 2003). We are currently investigating this phenomenon in BCY152.

### Two New Genes Involved in *C. albicans* Virulence

Two new genes, *MBF1* and *ZCF6*, were relevant for host systemic infection. Interestingly, and even if both genes were shown to be expressed *in vitro*, no *in vitro* phenotype was observed for either of the two mutants, except for a slight increase in the resistance of the *mbf1*Δ/Δ mutant to fluconazole. These results suggest that *MBF1* and *ZCF6* rather control host-specific features and should be further investigated.

Little is known about *ZCF6*, except that this gene encodes a putative TF with a zinc cluster DNA-binding domain. Although the absence of this gene does not affect the survival rates of infected animals, the proliferation of these fungal mutants is clearly and consistently impaired *in vivo* in both mammalian and insect models. Future experiments will help clarify the function of this gene, which might be specific for the *in vivo* host conditions, and determine *ZCF6*-regulated genes during systemic infections.

*MBF1* is an uncharacterized ORF with predicted sequence-specific DNA binding activity, reflecting the presence of a Cro/C1-type helix-turn-helix domain (IPR001387; Candida Genome Database, Gene Ontology Annotations for *MBF1*). A transcriptional coactivator has also been predicted based on gene orthology with *S. cerevisiae* (Candida Genome Database, Gene Ontology Annotations for *MBF1*). Concerning the function of *MBF1* in baker’s yeast, this gene has been annotated in the *Saccharomyces* Genome Database as a transcriptional coactivator linking the DNA-binding region of the Gcn4p and TATA-binding protein Spt15p (Takemaru et al., 1998), as a suppressor of frameshift mutations (Culbertson et al., 1982). The abundance of *S. cerevisiae* Mbf1 protein increases in response to DNA replication stress (Tkach et al., 2012; Saccharomyces Genome Database, Summary of MBF1/YOR298C-A).

## Conclusion

With the growing pressure for reducing the use of mammalian models, we conclude here that *G. mellonella* can be a valid model to substitute mouse pool infection experiments, since both approaches are reliable (50% phenotypic convergence as compared to the gold-standard mouse single strain infection). Thus, the two large scale screenings (*G. mellonella* single strain and mouse pool infections) are interesting approaches to predict mutants with a pronounced phenotype in the single strain infection mouse model. Accordingly, we were able in the present study to identify mutants with a known role in virulence and also a new factor, *MBF1*, which is annotated as a putative transcriptional coactivator. *MBF1* may therefore interact with several TFs, and therefore be implicated in the regulation of a number of different pathways. We are currently exploring the function of *MBF1* using *in vivo* transcriptional approaches to identify potential binding partners.

## Conflict of Interest Statement

The authors declare that the research was conducted in the absence of any commercial or financial relationships that could be construed as a potential conflict of interest.

## Footnotes

1 http://www.fgsc.net/

2 www.candidagenome.org
