# Supplementary material for: Examining the virulence of Candida albicans transcription factor mutants using Galleria mellonella and mouse infection models
Source: Front Microbiol. 2015 May 5;6:367. doi: 10.3389/fmicb.2015.00367 (PMC4419840; doi:10.3389/fmicb.2015.00367)
Supplement: Supplementary file 1 [file Table_1.DOCX]

**Supplementary Material**

**Examining the virulence of *Candida albicans* transcription factor mutants using *Galleria mellonella* and mouse infection models**

Sara Amorim-Vaz, Eric Delarze, Françoise Ischer, Dominique Sanglard*, Alix T. Coste

Institute of Microbiology, University of Lausanne and University Hospital Center, CH-1011 Lausanne, Switzerland

*Corresponding author: Institute of Microbiology, University Hospital Lausanne and University Hospital Center (CHUV), Rue du Bugnon 48, CH-1011 Lausanne, Tel: +41 21 314 40 83; Fax: +41 21 314 40 60; E-mail: Dominique.Sanglard@chuv.ch

**Supplementary Tables and Figures**

**Table S1**: **Primers used in this study**

| **Name** | **Sequence** |
| --- | --- |
| **Primers for construction of deletion and reversion cassettes** | |
| orf19.3294-5F-Apa | gcgaaagggcccCGTGATTTGACTTCAATGTCA |
| orf19.3294-5R-xho | cgcaaactcgagAAAAGAAAGTGTGAGTCAACG |
| orf19.3294-3F-SacII | cgcaaaccgcggGGGTTTCAATGATTGACGAAT |
| orf19.3294-3R-SacI | cgcaaagagctcATACCGCTGAAAAATGTTGAC |
| orf19.3294-XhoR | cgcaaactcgagATTCGTCAATCATTGAAACCC |
| orf19.1497-5F-Kpn | cgcaaaggtaccTCACACACACACTTTCTCTCT |
| orf19.1497-5R-xho | cgcaaactcgagTAACAGGTGGGGTTTGATCTT |
| orf19.1497-3F-sacII | cgcaaaccgcggTGCCAATTCAAAACAATTATGTG |
| orf19.1497-3R-SacI | cgcaaagagctcACTTCGATTCAAAACTGCAGA |
| orf19.1497-XhoR | cgcaaactcgagCTTCCAATGGCGAAAACAAAA |
| 2646bis-5for-kpn | gcgaaaGGTACCATGGATAAGACAAATAGTCC |
| 2646bis-5rev-xho | gcgaaaCTCGAGTATTGGCAAATATTGATGG |
| 2646bis-3for-sacII | gcgaaaCCGCGGATTTGTTGGTGATTTCCG |
| 2646bis-3rev-sacI | gcgaaaGAGCTCGAAGATCATTGAAATCACC |
| orf19.2646-5-for | cgcgaGGTACCTCAATCAAGCCTCCTGTACC |
| orf19.2646-5-rev | cgcgaCTCGAGATCGATTACTTTCTCCGTTG |
| orf19.2646-3-for | cgcgaCCGCGGAGGCTGCCAATTAAATAAC |
| orf19.2646-3-rev | cgcgaGAGCTCCTCATTATTAGGAGTTGC |
| orf19.2646-Rxho | cgcgaCTCGAGTGTACACAAAACTTAGAACC |
| **Sequencing Primers** | |
| orf19.2646-20 | CGGAGAAAGTAATCGA |
| orf19.2646+500 | CCGCCACCACCACCG |
| orf19.2646+100 | GTTGTCACCAACTAC |
| orf19.2646+1500 | ATCACCAGTGGCAAC |
| orf19.2646+2000 | TCCTATACTCCCGAA |
| orf19.2646+2500 | GTCTTGATGGGTGTG |
| orf19.2646+3000 | GTGTTGTACAATTGG |
| orf19.2646+3500 | TGCCATCAATACTAA |
| orf19.1497+500 | CAATAATAGTCTTCC |
| orf19.1497-70 | GTAAATCATCCTTATCC |
| orf19.1497+1200c | TCCATGAACTTTGGACA |
| orf19.1497+580c | GACAAATCATTCCTAGTG |
| orf19.3294-30seq | CGTTGACTCACACTTTCTTTT |
| **Primers (-F and -R) and probes (-P) for RT-qPCR** | |
| Act1-RT-F | ATAACGGTTCTGGTATGT |
| Act1-RT-R | CCTTGATGTCTTGGTCTA |
| Mbf1-RT-F | GCTGCCAGAAGAGCTGGTTT |
| Mbf1-RT-R | AACATCATCAGTTGCATCCAATTT |
| Zcf6-RT-F | CTGGAATCTGTTGTTGTTGCTAAAG |
| Zcf6-RT-R | GAGCCTCTTGGGTTTCCATTAGT |
| Act1-RT-P | CGGTGACGACGCTCCAAG |
| Mbf1-RT-P | ACGGAACTGCCAACACCAAGTCAAATC |
| Zcf6-RT-P | ATTAGAGACACAAAGCCACAATGGCAACAA |

**Table S2**: **Plasmids used in this study**

| **Plasmid** | **Origin** | **Description** | **Reference** |
| --- | --- | --- | --- |
| pAC244 | pBluescript II KS+ | Insertion of the KpnI-SacI fragment amplified with primers orf19.2646-5-for and orf19.2646-3-rev from SC5314 (orf19.2646) | This study |
| pAC245 | pAC244 | Ligation of the XhoI-SacII fragment of pSFS2A containing the *SAT1* cassette with fragment amplified with primers orf19.2646-5-rev and orf19.2646-3-for from pAC244 yielding the small deletion cassette for orf19.2646. | This study |
| pAC256 | pAC244 | Insertion of the KpnI-SacI fragment amplified with primers 2646bis-5for-kpn and 2646bis-3rev-sacI from SC5314 (orf19.2646) in pBluescript. Ligation of the XhoI-SacII fragment of pSFS2A containing the *SAT1* cassette with fragment amplified with primers 2646bis-5rev-xho and 2646bis-3for-sacII from pAC244 yielding the small deletion cassette for orf19.2646. | This study |
| pAC253 | pAC245 | Ligation of the KpnI-XhoI fragment of pAC245 with the fragment amplified with primers orf19.2646-5-rev and orf19.2646-Rxho | This study |
| pAC284 | pSFS2A | Insertion of the ApaI-XhoI fragment amplified with primers orf19.3294-5F-Apa and orf19.3294-5R-xho from SC5314 (5’-flanking region (500 bp) of orf19.3294) | This study |
| pAC286 | pAC284 | Insertion of the SacI-SacII fragment amplified with primers orf19.3294-3F-SacII and orf19.3294-3R-SacI from SC5314 (3’-flanking region (500 bp) of orf19.3294) yielding the orf19.3294 deletion cassette. | This study |
| pAC291 | pAC286 | Insertion of the ApaI-XhoI fragment amplified with primers orf19.3294-5F-Apa and orf19.3294-XhoR from SC5314 (orf19.3294) | This study |
| pSV1 | pSFS2A | Insertion of the KpnI-XhoI fragment amplified with primers orf19.1497-5F-Kpn and orf19.1497-5R-xho from SC5314 (5’-flanking region (500 bp) of orf19.1497) | This study |
| pSV3 | pSV1 | Insertion of the SacI-SacII fragment amplified with primers orf19.1497-3F-sacII and orf19.1497-3R-SacI from SC5314 (3’-flanking region (500 bp) of orf19.1497) yielding orf19.1497 deletion cassette. | This study |
| pSV4-1 | pSV3 | Insertion of the KpnI-XhoI fragment amplified with primers orf19.1497-5F-Kpn and orf19.1497-XhoR from SC5314 (orf19.1497 allele 1) | This study |
| pSV4-2 | pSV3 | Insertion of the KpnI-XhoI fragment amplified with primers orf19.1497-5F-Kpn and orf19.1497-XhoR from SC5314 (orf19.1497 allele 2) | This study |

**Table S3: Strains used in this study***

| **Strain** | **Parent** | **Genotype** | **Reference** |
| --- | --- | --- | --- |
| BCY31 | BWP17 | *RPS10*::CIp30 | (Vandeputte et al., 2011) |
| BCY82 | BWP17 | *cmp1*Δ::*hisG*/*cmp1*Δ::*hisG*, *RPS10*::CIp30 | (Vandeputte et al., 2011) |
| BCY11 | CAF4-2 | *RPS10*::CIp30 | (Vandeputte et al., 2011) |
| BCY13 | CAF4-2 | *cmp1*Δ::*hisG*/*cmp1*Δ::*hisG*, *RPS10*::CIp30 | (Vandeputte et al., 2011) |
| SC5314 | - | wild-type | (Gillum et al., 1984) |
| ACY296 | SC5314 | *zcf13*Δ::*FRT-SAT1*/*ZCF13* | This study |
| ACY297 | ACY296 | *zcf13*Δ::*FRT* /*ZCF13* | This study |
| ACY311 | ACY297 | *zcf13*Δ::*FRT-SAT1*/*zcf13*Δ::*FRT* | This study |
| ACY315 | ACY311 | *zcf13*Δ::*FRT*/*zcf13*Δ::*FRT* | This study |
| ACY317 | ACY315 | *zcf13*Δ::*FRT-SAT1*/*ZCF13* | This study |
| ACY360 | SC5314 | *mbf1*Δ::*FRT-SAT1*/*MBF1* | This study |
| ACY361 | ACY360 | *mbf1*Δ::*FRT*/*MBF1* | This study |
| ACY365 | ACY361 | *mbf1*Δ::*FRT-SAT1*/*mbf1*Δ::*FRT* | This study |
| ACY367 | ACY365 | *mbf1*Δ::*FRT*/*mbf1*Δ::*FRT* | This study |
| ACY370 | ACY367 | *mbf1*Δ::*FRT-SAT1*/*MBF1* | This study |
| SVY1 | SC5314 | *zcf6*Δ::*FRT-SAT1*/*ZCF6* | This study |
| SVY2 | SVY1 | *zcf6*Δ::*FRT*/*ZCF6* | This study |
| SVY3 | SVY2 | *zcf6*Δ::*FRT-SAT1*/*zcf6*Δ::*FRT* | This study |
| SVY4 | SVY3 | *zcf6*Δ::*FRT*/*zcf6*Δ::*FRT* | This study |
| SVY5-1 | SVY4 | *zcf6*Δ::*FRT-SAT1*/*ZCF6* | This study |
| SVY5-2 | SVY4 | *zcf6*Δ::*FRT-SAT1*/*ZCF6* | This study |

*All the BCY strains except BCY31 and BCY82 are listed in the Supplementary File S1

**Table S4: Efficacies of STM tag qPCR**

|  | STM6 | STM11 | STM20 | STM43 | STM209 | STM219 | STM224* | STM227 | STM232 | STM240 |
| --- | --- | --- | --- | --- | --- | --- | --- | --- | --- | --- |
| PCR efficacity () | 94.1 | 86.3 | 81.3 | 93.4 | 90.1 | 88.0 | 61.6 | 84.3 | 84.7 | 83.2 |
| SD | 9.9 | 11.5 | 14.3 | 5.6 | 10.6 | 32.9 | 11.0 | 8.9 | 16.6 | 19.3 |

*Efficacy of STM224 was too low (<65) and therefore results obtained with this tag were not included.

**Table S5: Proportion of similar fungal burden phenotypes observed between the different models of infections.**

|  | | Mutants with matching phenotypes (%) | | | | | | |
| --- | --- | --- | --- | --- | --- | --- | --- | --- |
|  |  | *G. mellonella* single strain infections ^a)^ | | | Mice single strain infections ^a)^ | | | CGD annotation of null mutants |
|  |  | exp1 | exp2 | **Merged** ^c)^ | exp1 | exp2 | **Merged** ^c)^ |  |
| Mice Pool infections | **Merged** ^c)^ | 27,7 | 27.2 | **21.5**  (25*;30.7^#^)^b)^ | 22.7 | 27.7 | **33.4**  (40*;50^#^) ^b)^ | **62.5** |
| *G. mellonella* single strain infections | exp1 |  | 45.2 | - | 33.3 | 33.3 | - ^d)^ | NA ^d)^ |
|  | exp2 |  |  | - | 53.3 | 46.6 | - | NA |
|  | **Merged** ^c)^ | - | - |  | - | - | **50** | NA |
| Mice single strain infections | exp1 | - | - | - |  | 61.1 | - | NA |
|  | exp2 | - | - | - |  |  | - | NA |
|  | **Merged** ^c)^ | - | - | - | - | - |  | NA |

^a)^ In *G. mellonella,* experiment 1 (exp1) is the merged of two experiments comprising 5 larvae each. Experiment 2 comprised 10 larvae tested once. Sub experiments 1 and 2 (exp1 and exp2) in mice comprised 10 mice tested once.

^b)^ Percentages between brackets indicate matching phenotypes considering mutants FB scores in mice pool experiments with * p-value ≤ 0.01, ^#^ FDR 0.05 corrected p-value.

^c)^ Merged data (bold type) comprised all experiments performed on one mutant. Comparisons between *G. mellonella* and mouse single strain infections, was done on 25 strains tested at least once in both organisms.

^d)^ NA: not applicable; -: not determined.

**Table S6: RT-qPCR detection of *MBF1* and *ZCF6* in *C. albicans* grown *in vitro* and *in vivo* (mouse kidneys or *G. mellonella*).**

|  |  | Experiment 1 | | | Experiment 2 | | |
| --- | --- | --- | --- | --- | --- | --- | --- |
|  |  | *ACT1* | *MBF1* | *ZCF6* | *ACT1* | *MBF1* | *ZCF6* |
| *In vitro* culture | Average CT | 25.38 | 25.58 | 31.54 | 24.74 | 25.63 | 33.50 |
| Infected mouse  (48 h pi) | Average CT | 28.83 | 30.46 | 36.30 | 28.19 | 30.62 | 40.46 |
|  | Fold change *in vivo* *vs in vitro* | 1.00 | 0.37 | 0.40 | 1.00 | 0.34 | 0.09 |
| Infected  *G. mellonella* (24 h pi) | Average CT | 32.91 | 33.47 | 36.79 | 31.98 | 33.84 | 37.28 |
|  | Fold change *in vivo* *vs in vitro* | 1.00 | 0.78 | 4.85 | 1.00 | 0.51 | 10.95 |
| Non-infected mouse | Average CT | N.D. | N.D. | N.D. | N.D. | N.D. | N.D. |
|  | Fold change *in vivo* *vs in vitro* | - | - | - | - | - | - |
| Non-infected  *G. mellonella* | Average CT | N.D. | N.D. | N.D. | N.D. | N.D. | N.D. |
|  | Fold change *in vivo* *vs in vitro* | - | - | - | - | - | - |

The expression level of *ACT1* was used for normalization. Cycle threshold (CT) values shown represent the average of two technical replicates of two biological duplicates (therefore 4 values in total), and the experiment was performed twice. Fold change values were calculated via the 2^ΔΔCT^ formula. N.D.: not detected.

**Figure S1**: Results of mice pool infections with CFU quantification in the kidneys at 3 dpi. Barcodes specific for each mutant were used for qPCR quantification. For each mutant, a fungal burden score was calculated and normalized against the WT strain carrying the same barcode (see Material and Methods). Statistical analyses were performed by a Mann-Whitney test to assess fungal burden score differences relatively to the wild type strain carrying the same barcode. Only mutants that were scored in at least 3 mice were taken into account. Mutants displaying less than 3 scores were considered as non-detected. Due to a problem with the detection of the STM224 barcode, strains carrying this tag were considered as non-tested.


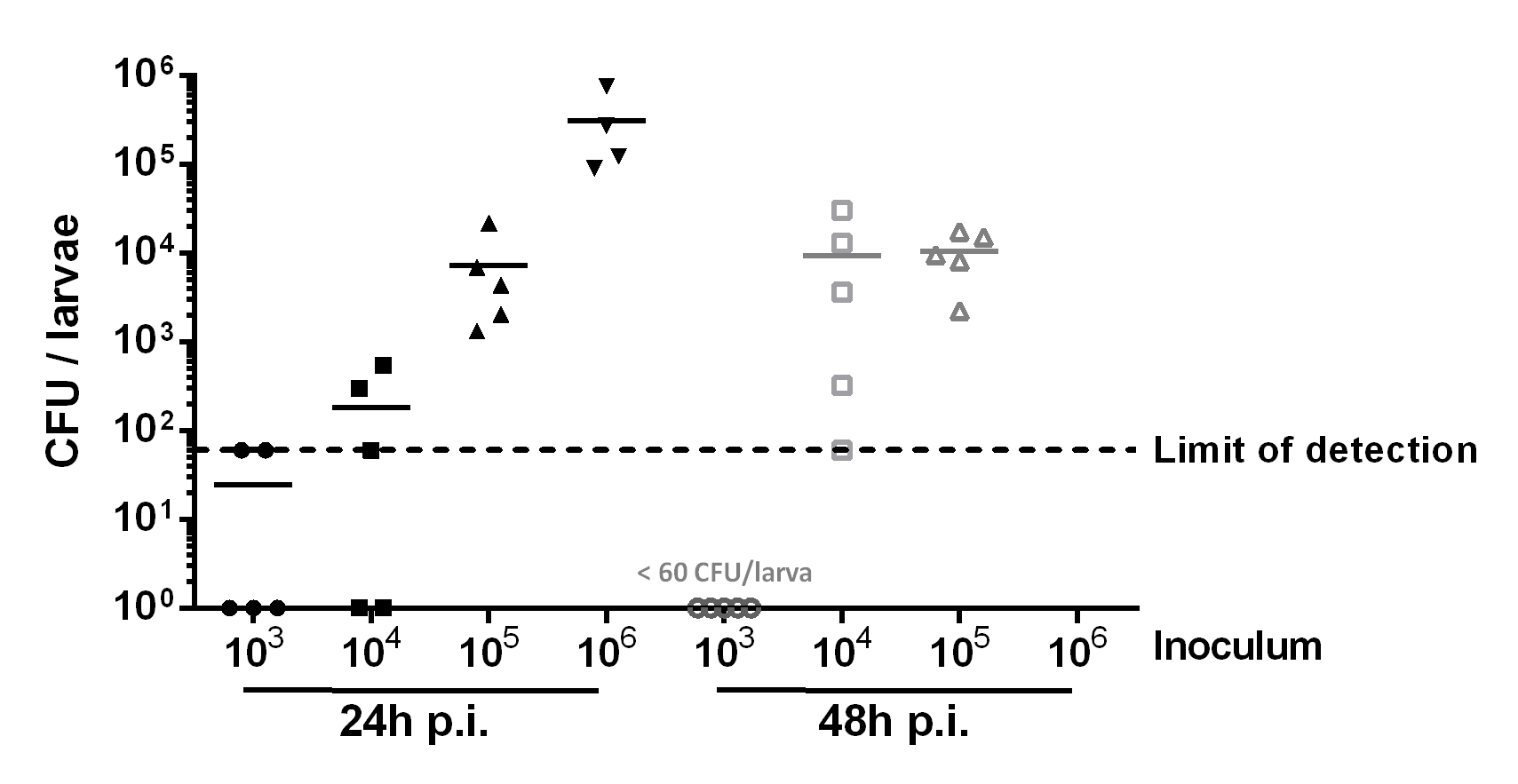


**Figure S2**: Determining optimal *C. albicans* inoculum and time post-infection (p.i.) for *Galleria* fungal burden quantification. Limit of detection is 60 CFU/larva. Larvae infected with 10^6^ *C. albicans* cells were all dead 48h p.i. Clear dose-response can be visualized only at 24h p.i. The inoculum of 10^5^ *C. albicans* cells allows visualization of fungal burdens higher or lower the wild type strain without resulting in the death of the larvae or in CFU values below the limit of detection.

**Figure S3**: Growth curves of the newly constructed mutants, and corresponding revertants, of genes *MBF1*, *ZCF6* and *ZCF13*. Graphs represent means of biological duplicates with technical duplicates.


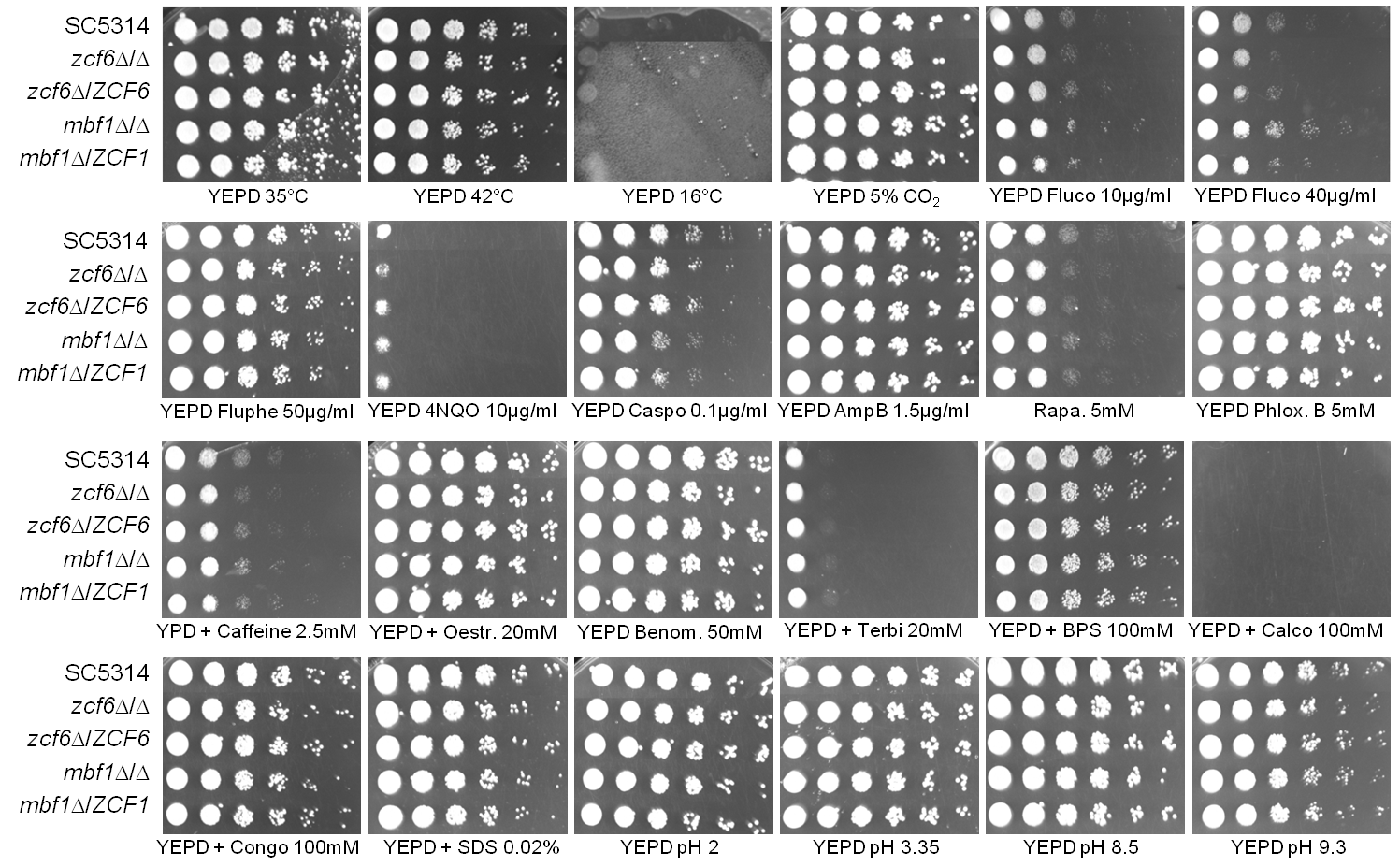

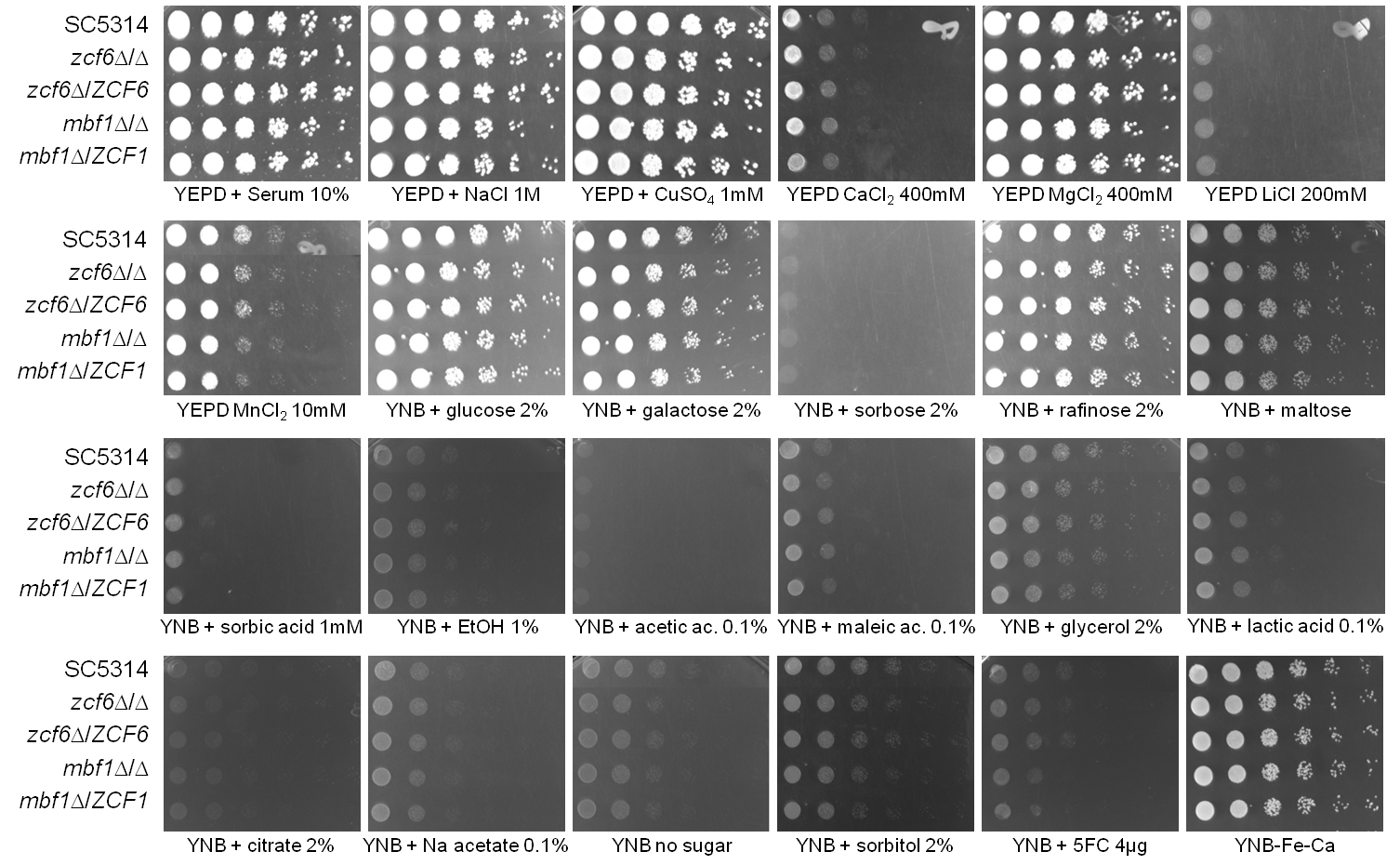


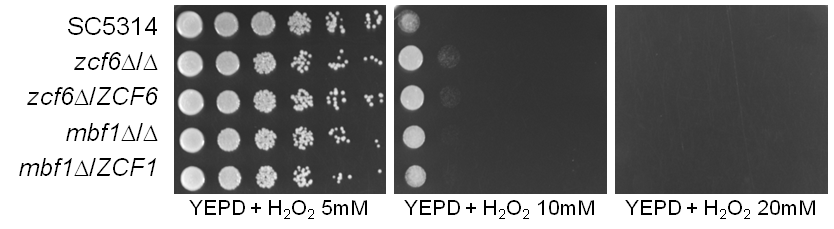


**Figure S4:**  *In vitro* phenotype of *mb1f*Δ/Δ and *zcf6*Δ/Δ strains, as well as the corresponding revertant strains, as compared to the wild type, after 24h of incubation. YEPD – yeast extract peptone dextrose. Fluco – fluconazole. Fluphe – fluphenazine. 4NQO - 4-Nitroquinoline 1-oxide. Caspo – casponfungin. AmpB – amphotericin B. Phlox. B - . Rapa – rapamycin. Oestr – oestrogen. Benom - benomyl . Terbi – terbinafine. BPS - bathophenanthroline disulfonate. Calco – calcofluor white. Congo – congo red. SDS – sodium dodecylsulfate. EtOH – ethanol. YNB – yeast nitrogen base. YNB-Fe-Ca – YNB minus iron and calcium ions. 5FC - 5-fluorocytosine.

**References**

Gillum, A.M., Tsay, E.Y., and Kirsch, D.R. (1984). Isolation of the Candida albicans gene for orotidine-5'-phosphate decarboxylase by complementation of S. cerevisiae ura3 and E. coli pyrF mutations. *Mol Gen Genet* 198**,** 179-182.

Vandeputte, P., Ischer, F., Sanglard, D., and Coste, A.T. (2011). In Vivo Systematic Analysis of Candida albicans Zn2-Cys6 Transcription Factors Mutants for Mice Organ Colonization. *PLoS ONE* 6**,** e26962. doi: 10.1371/journal.pone.0026962.t004.
